# Advancing Tobacco Cessation in LMICs

**DOI:** 10.3390/curroncol29120713

**Published:** 2022-11-23

**Authors:** Abhishek Shankar, Mark Parascandola, Pirabu Sakthivel, Jagdish Kaur, Deepak Saini, Naveen Prabhu Jayaraj

**Affiliations:** 1 Department of Radiation Oncology, All India Institute of Medical Sciences, Patna 801507, India; 2 Research and Training Branch, Center for Global Health, National Cancer Institute, Bethesda, MD 20892, USA; 3 Department of ENT & Head-Neck Surgery, KMCH Institute of Health Sciences and Research, Coimbatore 641014, India; 4 Department of Healthier Populations and Non-Communicable Diseases (NCDs), WHO Regional Office for South-East Asia, New Delhi 110011, India; 5 Materia Medica Department, Lal Bahadur Shastri Homoeopathic Medical College and Hospital, Prayagraj 211013, India; 6 Department of Community Medicine, Karpagam Faculty of Medical Sciences and Research, Coimbatore 641032, India

**Keywords:** tobacco, tobacco cessation, smoking, LMIC, tobacco-cessation intervention

## Abstract

Tobacco kills more than 8 million people worldwide every year. Over 80% of the world’s 1.3 billion tobacco users live in low- and middle-income countries (LMICs), where the future burden is projected to grow. At the same time, progress in tobacco control has not advanced as far as in many LMICs. In particular, the implementation of tobacco-cessation programs and interventions remains limited. The bulk of the evidence for tobacco-cessation interventions comes from high-income countries and may not reflect the context in LMICs, particularly as resources and training for tobacco cessation are limited. This paper summarizes the current evidence for tobacco-cessation interventions in LMICs and highlights some key challenges and research gaps. Overall, there is a need to build capacity for locally relevant research and implementation science to support tailored cessation interventions and strategies for LMICs.

## 1. Background

Tobacco use is the leading cause of preventable death globally [1]. Over 80% of the world’s 1.3 billion tobacco users live in low- and middle-income countries (LMICs), and it is projected that, if current trends continue, by the year 2030, 70% of the estimated 10 million smoking-related deaths will occur in LMICs [2]. While in many high-income countries, smoking prevalence has been decreasing in response to decades of tobacco-control efforts, this is not the case in many LMICs [3]. Moreover, as in most high-income countries, there are substantial disparities in tobacco use and its health consequences among LMICs, as tobacco use tends to be greater among men, those with lower education, less household wealth, living in rural areas, and higher age [4,5].

As of 2022, the vast majority of countries (182) are parties to the WHO Framework Convention on Tobacco Control (FCTC) [6]. The WHO Global Action Plan for the Prevention and Control of Non-Communicable Diseases, endorsed by the World Health Assembly, set a target of a 30% relative reduction in the prevalence of current tobacco use by 2025 [7]. An evidence-based package of tobacco-control strategies already exists in the form of the WHO MPOWER strategies (Monitor tobacco use, Protect from tobacco smoke, Offer help to quit, Warn about dangers, Enforce bans on promotions, and Raise taxes). WHO reported that as of 2019, 65% of the world’s population and 61% of those living in LMICs were covered by at least one MPOWER measure (not including ‘Monitor’) [8]. As a result of the FCTC, many LMICs have adopted national policies with the aim of creating awareness about the harmful effects of tobacco consumption and reducing the production and supply of tobacco products, including helping people quit tobacco use with evidence-based strategies.

While the reach of the FCTC and related MPOWER measures have expanded over the past decade, full implementation of the treaty remains a challenge, particularly in many LMICs. As of 2020, comprehensive tobacco-cessation services are in place for 2.5 billion people in 26 countries, or 32% of the world’s population. The number of countries adopting comprehensive tobacco-cessation measures lags behind the other MPOWER measures, with only seventeen high-income countries and nine middle-income countries offering comprehensive cessation support. No low-income countries currently offer best-practice services. Globally, almost all high-income countries (89%) offer at least partial coverage of the cost of cessation services. Most middle-income countries (72%) do the same, while 18% of low-income countries offer some cost coverage for services. Additionally, while over 80% of FCTC countries impose a tax on cigarettes, only a fraction of countries (14%) meet FCTC tobacco tax guidelines [8,9]. Tobacco-control efforts may face unique challenges in LMICs, given limited resources, competing for health priorities, and diverse cultural and social contexts [10]. The diversity of types of tobacco products in use around the world, including cigarettes, chewing tobacco, bidis, hookah/sheesha, and novel tobacco products such as HTPs (heated tobacco products), creates additional challenges for tobacco-control efforts [11,12]. Traditional tobacco products in some LMICs may be produced in a local ‘cottage industry’ or prepared for the user by a street vendor, making it difficult to monitor or regulate these products [13]. To be effectively implemented, tobacco-control interventions and policies may need to be adapted to LMIC settings in terms of their unique social, cultural, political, economic, and regulatory contexts; tobacco product availability and use patterns; and tobacco industry influence, among other factors [14]. 

Support for tobacco cessation is one key component of the WHO FCTC, MPOWER measures, and Best Buys that remains underutilized. Although many countries offer some form of tobacco-cessation support in some settings, the accessibility and coverage of such services are often limited, as countries may lack the infrastructure needed to offer widespread population-level cessation support to the majority of tobacco users [15]. Although many countries report offering cessation services, these services do not necessarily meet WHO guidelines for WHO FCTC implementation at the national level [16,17]. Persisting tobacco consumption in LMICs and the unique challenges of implementing tobacco cessation in these regions mandate a specific focus on the efficacy and implementation of interventions in LMICs. In this paper, we focus on tobacco-cessation programs for LMICs.

## 2. The Landscape of Tobacco-Cessation Measures

For those making decisions around health programs and policies, it is essential to have evidence regarding which interventions are effective in the prevention and control of tobacco use. 

### Literature Search and Methodology

We reviewed retrospectively published academic literature in PubMed, Cochrane Reviews, Scopus, Web of Science, and Google Scholar until September 2022 and compiled the experience working on smoke-free implementation in LMICs. We searched for combinations and suffix variations of (‘Tobacco cessation’ or ‘control’ or ‘quitting’) and (‘tobacco’ or ‘smoking’) and (‘LMICs’). Those articles that addressed tobacco cessation in an LMIC setting and provided data or described attempted solutions or lessons learned were included. Effective implementation of policies related to reduction in demand and supply of tobacco, as advocated by WHO FCTC and MPOWER package, generates increased demand for quitting tobacco use. Table 1 below describes evidence-based policies to reduce the demand and supply of tobacco. Sakthivel et al., drawing on experience from India and other LMICs, classified tobacco-control and -cessation activities in an innovative “A to Z” way under policy (government) measures, pharmacological and non-pharmacological measures (Table 1) [18]. We apply this classification to describe the evidence for tobacco-cessation approaches in LMICs and organize the discussion that follows.

## 3. Policy Measures

Evidence-based tobacco-control policies and programs implemented by the government help promote cessation, even when they are not specifically targeted at treating tobacco dependence by creating a conducive environment for cessation. Tobacco-control policies, including raising taxes and smokefree laws, reduce smoking and increase quit attempts at the population level [19]. Mass media campaigns have been used since the 1970s to reduce tobacco use at population level, educate about the harms of smoking, change smoking attitudes and beliefs, increase quitting intentions and quit attempts, and reduce adult smoking prevalence. A Cochrane review by Bala et al. found that mass media campaigns, when included in comprehensive tobacco-control programs, can be effective in changing smoking behavior in adults [20]. Additionally, evidence from a variety of countries demonstrates that tax policies to increase the price of tobacco products and comprehensive smoke-free policies prohibiting smoking in workplaces, and public places, including bars and restaurants, can encourage more tobacco users to quit [13]. 

A recent literature review by Byron et al. found that where smoke-free policies exist in LMICs, they reduce smoke exposure, increase quit intentions, and potentially decrease youth smoking initiation. However, the actual implementation of smoke-free policies in LMICs has been mixed in various regions of the world [21]. Support for tobacco cessation needs to be comprehensive and available at population level. 

## 4. Pharmacological Measures

While a substantial body of evidence exists to support a range of pharmacological interventions for tobacco cessation, most of this evidence comes from HICs and is only partly applicable to many LMICs [21,22,23,24,25]. As more studies have been conducted in LMICs, more recent reviews have added further support, though the evidence base remains limited. Akanbi et al. conducted a meta-analysis of 24 tobacco-cessation RCTs in LMICs limited to those with at least 6 months of follow-up; 4 trials that compared nicotine replacement therapy to placebo found NRT improved cessation rates (n: NRT 546, control 684, OR = 1.76, 95% CI = 1.30–2.77, *p* < 0.001) [25]. A recent scoping review by Kumar et al. identified 92 tobacco-cessation RCTs conducted across 16 LMICs, with the majority from India and China. However, they described the evidence as weak in quality and severely limited. Most RCTs were limited to evaluating the effects of different forms of counseling rather than targeted behavioral and pharmacological interventions [26]. 

Studies across a number of LMICs have shown benefits from nicotine replacement therapy and other pharmacologic treatments in isolation or in conjunction with behavioral therapy for cessation compared to placebo. A randomized control trial (RCT) conducted in India by Singh et al., using bupropion, had a significant difference in the 7-day point prevalence abstinence rate at the end of week 2 (*p* = 0.04) [27]. Sharma et al. used varenicline for RCT among smokeless tobacco users receiving behavioral counseling in India. Self-reported abstinence was significantly greater for varenicline (43%) versus placebo (31%; adjusted odds ratio = 2.6, 95% CI = 1.2–4.2, *p* = 0.009) [28]. Heydari et al. in Iran compared three interventions among smokers: Brief counseling vs. Nicotine patch vs. Varenicline. They employed brief counseling sessions and nicotine patches 15 mg/daily for 8 weeks, or one 0.5 mg varenicline pill daily dosed up over 8 weeks. Follow-up at a year showed 6.6% of the first group, 25% of the second group, and 32.6% of the third group remained smoke-free [29]. Koegelenberg et al. in South Africa, in their RCT of 435 patients, concluded Varenicline in combination with NRT was more effective than varenicline alone at achieving tobacco abstinence at 12 weeks (end of treatment) and at 6 months [30]. 

However, despite significant efforts, several factors have limited the reach of tobacco-cessation treatment in LMICs. Potential barriers include a perceived lack of interest from patients, continued tobacco use among medical practitioners, lack of familiarity or training related to pharmacological and behavioral treatments, and lack of government support [31]. Additionally, the cost may be seen as a barrier to government support for cessation clinics and the provision of medication, particularly when faced with competing health priorities. However, tobacco-cessation support remains highly cost-effective, even in low-resource settings. Moreover, as the costs of smoking-related morbidity and mortality increase in LMICs, as projected, the benefit from investing in cessation programs will increase as well [13]. 

## 5. Non-Pharmacological Measures

In addition to pharmacological treatments, behavioral therapies and other non-pharmacological interventions can also be effective in helping smokers to quit. There is substantial evidence for individual, group, and telephone counseling, telephone quitlines, and mass media campaign strategies [32]. Evidence for the effectiveness of these interventions from LMICs remains relatively limited. As more studies have been conducted in LMICs, more recent reviews have added further support, though the evidence base remains limited. A 2012 systematic review of 45 pharmacologic and behavioral trials conducted in LMICs found limited evidence that nicotine replacement therapy (NRT) and bupropion may help smokers to stop smoking and probably reduce smoking rates [24]. 

Several studies conducted in LMICs provide noteworthy findings. The ESCAPE study conducted in Thailand employed a multi-component intervention consisting of regular patient motivation by the same nurse over a 3-month period, a monthly Smokerlyzer test for 3 months, continual assistance from a trained family member, use of a smoking-cessation diary, and access to optional nicotine replacement chewing gum. At the end of one year, the intervention arm participants achieved a significantly higher smoking cessation rate than the control arm (25.62% vs. 11.32%), which received only brief counseling and casual follow-up, suggesting that intensive tobacco-cessation interventions can be effective within primary health care settings in an LMIC [33]. Otero et al. in Brazil compared cognitive behavioral therapy (CBT) sessions with and without nicotine replacement therapy for smoking cessation. They found that the use of NRT along with CBT increased abstinence, but also that a greater number of CBT sessions increased abstinence, independent of NRT, suggesting a dose–response relationship [34]. An RCT by Sorensen et al. in India used a cluster-randomized design to test the intervention, which comprised educational efforts, tobacco-control policies, and cessation support targeting the t school teachers reported a 30-day abstinence rate of 50% and a 6-month abstinence rate of 20% among the teachers, who may go on to serve as role models for tobacco control in their communities [35]. 

In an RCT in Vietnam, Shelley and colleagues established the effectiveness of a multi-component strategy model (4As—Ask about tobacco use, Advise to quit, Assess readiness, and Assist with brief counseling plus more intensive counseling by health workers) in enhancing tobacco dependence treatment rates. However, access to cessation treatment was limited to major health centers. After studying barriers and facilitators to delivering cessation treatment, they tested a strategy to train community health workers to provide counseling and conduct follow up with patients in their local communities [36]. Strategies involving “task shifting” provide an important avenue to scaling up cessation services. 

Integrating tobacco cessation into existing health systems represents another important strategy for increasing access and reach of cessation services. Brief advice is provided to TB patients using tobacco under integrated TB-tobacco programs. A study in India showed that more than 67% of TB patients who used tobacco were able to quit tobacco use at the completion of TB treatment when provided brief advice by trained DOTS providers [37].

Digital technologies (such as mobile phone-based tools) also offer novel opportunities for increasing the reach of cessation services, especially for challenging or low-resource settings in LMICs.In recent years, cell phone penetration has increased to over 90% in LMICs, a figure that is higher than the global average [38]. There is now moderate evidence that automated-text-message-based smoking cessation interventions result in higher quit rates than minimal smoking-cessation support [39]. An RCT by Ybarra et al. in turkey employed a text-messaging-based smoking cessation program for adult smokers and found that employing mobile technologies may be able to affect quitting rates in environments with high smoking prevalence [40]. Another RCT by Liao et al. compared 12 weeks of the “Happy Quit” text messaging intervention, using either high-frequency messaging (HFM) or low-frequency messaging (LFM) intervention in a control group in China; they found that both the high- and low-frequency message conditions increased cessation [41]. The mCessation program implemented by the Government of India to help tobacco users quit by using mobile-phone-based messages motivated a large number of subscribers to attempt to quit tobacco use and for many to achieve 30-day quit status [42]. National toll-free quitlines have been established by many countries to support tobacco cessation. In resource-limited settings, quitlines can play a greater role in helping people quit smoking as part of a comprehensive approach [43].

While anti-tobacco mass media campaigns have been employed in LMICs as well, there are limited data on their impact on cessation. In India, a national television and radio campaign targeting smokeless tobacco users was associated with increased awareness and cessation-oriented intentions and behaviors [44]. However, there is still a need for more studies to be conducted in LMICs.

## 6. Discussion

There are a number of challenges to implementing tobacco-cessation activities in LMICs, including a diversity of tobacco products and practices, limited capacity and resources, competing health priorities, and limited attention to cessation/dependence. One important barrier is the fact that most strategies are adopted directly from HICs which may not be feasible and applicable to the context of LMICs due to differences in social, economic, cultural, environmental, and political factors. Most LMICs lack the decades of experience and capacity that many HICs had acquired over the years to develop, evaluate, and implement tobacco-control interventions. High tobacco use among health professionals in some LMICs is an additional barrier to sending the right message to the community. Distinct patterns of tobacco use, such as the high prevalence of smokeless tobacco use in South Asia, may require different approaches; pharmacotherapies effective for treating cigarette smokers have had less success for smokeless tobacco users [45]. There is a need for further research on interventions tailored to and evaluated in diverse LMICs, including the development of low-cost cessation interventions and integration of cessation services into health systems [46]. 

Going forward, concepts and methods from the field of implementation science may be especially important for advancing tobacco cessation in LMICs. Tools used in implementation science include (i) models that describe and guide the process of translating research into practice, (ii) determinants frameworks that describe factors that influence implementation outcomes, and (iii) evaluation frameworks to assess implementation success [47]. Implementation science can inform the adaptation and testing of interventions and implementation strategies in LMIC settings with the goal of increasing the reach and impact of tobacco-cessation measures and ultimately reducing the burden of tobacco-related morbidity and mortality [46]. Such research requires the generation and use of local, contextual evidence from the setting in which the intervention is to be implemented [48]. 

## 7. Conclusions

While a few studies conducted in LMICs have shown positive results for pharmacologic and behavioral interventions for smoking cessation, the evidence base outside HICs remains limited to informing the implementation of tobacco-cessation interventions across diverse LMIC settings. In addition to addressing specific research needs, it is critical to advance research capacity in LMICs to develop research that is locally relevant and contextually informed. Likewise, sensitization of health professionals to their role in tobacco cessation and the provision of adequate training are essential to shift the culture around tobacco use in many settings. Under current trends, the burden of tobacco use in LMICs will continue to grow, but this outcome is not inevitable. Continued research and targeted action to reduce tobacco use, including cessation efforts along with strong tobacco-control policies, can alter future outcomes.

## Figures and Tables

**Table 1:** Classification of tobacco-control activities.

Policies to Reduce Demand and Supply of Tobacco	Tobacco-Cessation Activities	
(Government) Policy Measures	Pharmacological Measures	Non-Pharmacological Measures
Regular monitoring of tobacco use, prevalence, and tobacco-control policy implementation Comprehensive smokefree laws Selective ban on smokeless tobacco products Ban on tobacco advertising, promotion, and sponsorship (TAPS) Restricting tobacco sale to and by minors Raising tax on tobacco Anti-tobacco mass media campaigns Warning labels on tobacco products	Nicotine Replacement therapy Non-nicotine medications	Brief advice—A” and “R” Technique (The five A’s: Ask, Advise, Assess, Assist and Arrange and five R’s: Relevance, Risk, Rewards, Repetition, and Roadblocks) - Tailored interventions for high-risk groups - Family or group-based programs - Incentive-based cessation programs - Mobile apps to aid cessation (WHO QuitTobacco App) - mCessation- WHO-ITU Be Healthy Be -Mobile mobile phone-based cessation program - Florence: the digital health worker to support quitting tobacco - Toll-free Quitline

## Data Availability

Not applicable.
